# Association Between Dietary Quality and Postpartum Depression in Lactating Women: A Cross-Sectional Survey in Urban China

**DOI:** 10.3389/fnut.2021.705353

**Published:** 2021-08-26

**Authors:** Chenlu Yang, Ai Zhao, Hanglian Lan, Zhongxia Ren, Jian Zhang, Ignatius Man-Yau Szeto, Peiyu Wang, Yumei Zhang

**Affiliations:** ^1^Department of Nutrition and Food Hygiene, School of Public Health, Peking University, Beijing, China; ^2^Vanke School of Public Health, Tsinghua University, Beijing, China; ^3^Inner Mongolia Dairy Technology Research Institute Co.Ltd., Hohhot, China; ^4^Yili Maternal and Infant Nutrition Institute, Inner Mongolia Yili Industrial Group Co.Ltd., Hohhot, China; ^5^Department of Social Medicine and Health Education, School of Public Health, Peking University, Beijing, China; ^6^Beijing Key Laboratory of Toxicological Research and Risk Assessment for Food Safety, School of Public Health, Peking University, Beijing, China

**Keywords:** diet quality, postpartum depression, diet balance index, lactating women, Chinese dietary guidelines

## Abstract

**Background:** Evidence on the effects of dietary quality on the risk of postpartum depression in the Chinese population is limited. This study aimed to examine the association between dietary quality and postpartum depression in Chinses lactating women.

**Methods:** A total of 939 participants from 10 cities were included in this analysis. A one-time 24-h dietary recall was used to obtain the data on food consumption and dietary quality was assessed based on Diet Balance Index. The Edinburgh postnatal depression scale was considered at a cutoff point of 10 to detect postpartum depression. Poisson regression models were used to explore the association of dietary quality with postpartum depression.

**Results:** Depressed women tended to have a more inadequate intake of vegetables and have more insufficient food variety. The median (25th, 75th) of the overall high bound score (HBS), low bound score (LBS), and diet quality distance (DQD) was 9 (5, 14), 30 (25, 37), and 40 (34, 47), respectively. Compared with subjects with the lowest quartile of LBS, those with the highest quartile of LBS had a higher risk of postpartum depression [adjusted prevalence ratio (aPR), 1.08; 95% confidence interval (95% CI), 1.01, 1.15; P for trend, 0.043]. We also observed a significant association between DQD and postpartum depression (Q4 vs. Q1: aPR, 1.07; 95% CI: 1.00, 1.14; P for trend, 0.036).

**Conclusion:** Poor dietary quality was associated with postpartum depression in Chinese lactating women.

## Introduction

Postpartum depression is a widely recognized public health concern that has important implications for the health and well-being of many new mothers, their infants, and their families (1, 2). Depressive symptoms may include extreme sadness, fatigue, anxiety, crying, irritability, and changes in lifestyle habits (3). A meta-analysis has reported that pooled prevalence of postpartum depression was 14.8% and showed a significant increasing trend in the last decade in Mainland China (4). There may be numerous mechanisms mediating the development of a typical pathophysiological signature associated with postpartum depression (5). Still, the etiology of postpartum depression has not been fully cleared. Even worse, some women may be unwilling to take pharmacological treatments, because they concern about the transmission of antidepressant medication to their infants if they are breastfeeding (6). Therefore, the efforts toward the identification of risk factors are crucial for both maternal and children's health and well-being.

Dietary quality may be a modifiable risk factor for depression because some nutritional factors may modulate the potential biological pathways related to mental disorders, such as inflammation, oxidative stress, the gut microbiome, epigenetic modifications, and neuroplasticity (7). A healthy dietary pattern, which was characterized by fruits, vegetables, whole grains, and fish, may decrease the risk of depression (8, 9). However, the associations of dietary quality with postpartum depression have primarily been explored in western countries (10–13), and results were inconsistent. Researches among Chinese lactating women are less. Despite the knowledge that lactating women require varied diets and increased nutrient intake, Chinese lactating women tend to be more prone to poor dietary quality, especially for the women in the puerperium. Chinese traditional beliefs about the importance of “*Yin-Yang* balance” affect the daily food practices of lactating women. Childbirth is believed to disturb the “*Yin-Yang* balance,” and different food characteristics (such as “hot” and “cold”) can alter the “*Yin-Yang* balance” in the body (14–16). Usually, the “cold” foods were related “Yin.” Therefore, lactating women should avoid “cold” foods to maintain body warmth and restore maternal “*Yin-Yang* balance.” For example, fresh fruits or vegetables, even seafood, are not highly recommended because most of these foods are considered to have “cold” properties. In addition, lactating women are encouraged to consume more certain foods, such as animal products, red sugar, even rice wine, because these foods have been traditionally regarded as beneficial to maternal health and breast milk quantity and quality. However, these dietary restrictions are not aligned with nutritional needs and may cause some common postpartum symptoms. For example, a decreased intake of fruit and vegetables and excessive sugar intake are associated with a high incidence of constipation, hemorrhoids, and oral problems (17).

The objective of the current study was to explore the potential associations between dietary quality and postpartum depression.

## Methods

### Participants

The data were collected as part of the Young Investigation (YI Study), which was a cross-sectional survey on health and nutrition status of pregnant women, lactating women, young children aged 0–3 years from 2019 to 2020. Two first-tier cities (Beijing and Guangzhou), three new first-tier cities (Suzhou, Chengdu, and Shenyang), three second-tier cities (Ningbo, Lanzhou, and Nanchang), one third-tier city (Hohhot), and one fourth-tier city (Xuchang) were selected. One hospital or one maternal and child health care center was selected in each city. The target was to recruit at least 90 lactating women in each city, and lactating women were conveniently recruited according to their visiting time until the number of participants satisfied the sample size. For lactating women, the inclusion criteria were healthy women in the first year postpartum, aged between 20 and 45 years, with singleton delivery, no smoking or alcohol abuse, without mastitis or any infectious diseases, and without cardiovascular or metabolic diseases. We excluded those participants with missing data or extreme outliers for key variables under the purpose of this report. Finally, 939 participants were enrolled in the current study.

### Data Collection and Measures

The investigation was conducted using face-to-face interviews. All interviewers were subject to unified training before starting the investigation.

Postpartum depression was assessed by the Edinburgh Postnatal Depression Scale (EPDS). The EPDS included 10 items using 4-point response options ranging from 0 to 3 to capture symptoms in the previous seven days. The total score ranged from 0 to 30, with higher scores indicating greater severity of depression. The cutoff scores of ≥10 were used to classify non-depressed and depressed mothers, respectively, and the cutoff scores of ≥13 were used to further classify mildly depressed and moderate or severe depressed mothers, respectively, among the depressed mothers (18, 19).

A one-time 24-h dietary recall (24HDR) was used to obtain the data on food consumption over the previous 24 h to the investigation. With the help of trained interviewers, participants were asked to recall all food, beverages, and condiments consumed individually over the previous 24 h to the investigation. Standard-sized bowls, standard-sized teaspoons, and illustrated photos of food items were shown to help participants to assess quantities (20). Total energy intake was calculated based on the Chinese Food Composition Table coupled with the nutrition information packaging (21). In China, Diet Balance Index (DBI) was recommended to assess human diet quality. It can measure the dietary quality as a whole, and reflect not only insufficient dietary intake but also excessive dietary intake (22, 23). DBI was established based on the Chinese dietary guidelines. It was firstly released in 2005 and has been updated twice, with DBI_16 being the current measure (24–26). DBI_16 was based on current Chinese dietary guidelines and Food Guide Pagoda (2016), and has more specific energy assignment levels that comprehensively reflect the dietary quality of the population. DBI_16 could be minor adjusted to meet the needs of different populations based on specific dietary guidelines, and a previous study has proved that adjusted DBI was a reliable tool to measure the diet quality among lactating women (27). In this study, DBI comprised eight components, namely (range of values) (1) cereals (−12~12); (2) vegetables (−6~0), fruits (−6~0); (3)dairy (−6~0), soybean (−6~0); (4) animal foods (−4~4 for meat and poultry, −4~0 for fish and shrimp, −4~4 for eggs); (5) empty energy food (0~6 for cooking oil, 0~6 for alcohol); (6) salt (0~6); (7) food variety (−12~0); and (8) water (−12~0). A score of 0 for each component indicated that the recommended intake had been met. The positive or negative scores indicated that recommended level was exceeded or not met, respectively. The specific calculating methods of each component are given in Supplementary Table 1. After calculating the scores of each component, high bound score (HBS), low bound score (LBS), and diet quality distance (DQD) were calculated. The HBS referred to the sum of all positive scores, indicating excessive food intake (range: 0~38). The LBS referred to the sum of the absolute values of all negative scores, indicating insufficient food intake (range: 0~72). DQD referred to the sum of the absolute values of both positive and negative scores, indicating imbalanced food intake (range: 0~90). The larger values for HBS, LBS, and DQD indicate more inferior diet quality. HBS, LBS, and DQD were further divided into four levels (excellent or good, mild poor, moderately poor, and severe poor) according to 0~40%, 40~60%, 60~80%, and 80~100% of total scores, respectively.

Essential characteristics of the lactating women were collected, including age, education, family monthly per capita income (Chinese yuan), puerperium (within 6 weeks after delivery) or not, and primiparas or not. Weight and height were measured on the day of investigation, and body mass index (BMI) was coded into three categories: underweight or normal (BMI <24 kg/m^2^), overweight (24 ≤ BMI <28 kg/m^2^), and obese (BMI ≥ 28 kg/m^2^) (28). Based on the short version of the International Physical Activity Questionnaire (IPAQ), metabolic equivalent of energy (MET) hours per week were calculated, and the physical activity of participants was equally divided into three groups: Low, medium, and High (29). The status of husband or partner, and parent support were self-reported by lactating women (rarely, sometimes, or always).

### Statistical Analyses

The statistical analysis was performed by and SPSS 26.0. All non-normally distributed continuous variables are presented as median (25th, 75th). All categorical variables are presented as percentages. First, chi-square tests were performed to compare the characteristics of lactating women by depression status. Second, Mann-Whitney U or Kruskal-Wallis H tests were performed to compare the HBS, LBS, and DQD by different characteristics of lactating women. Third, Spearman correlation analyses were performed to explore the associations of total scores of EPDS with HBS, LBS, and DQD. Mann-Whitney U tests were performed to compare each food component, HBS, LBS and DQD of lactating women by depression status. Fourth, poisson regression models were performed to explore the associations of HBS, LBS, and DQD with postpartum depression, and prevalence ratios (PRs) and their respective 95% confidence intervals (95%CIs) were estimated.

## Results

As shown in Table 1, the overall proportion of postpartum depression was 33.9%, which was comprised of mild depression (17.7%) and moderate or severe depression (16.2%). Depressed women tended to be younger, less educated, primiparas, and with less husband or partner support.

**Table 1:** Characteristics of lactating women by depression status.

**Variables**		**Total**	**Non-depression**	**Mild depression**	**Moderate or severe depression**	***P*^a^**
N	–	939	621	166	152	–
Age (years)	≤ 30	472 (50.3)	290 (46.7)	87 (52.4)	95 (62.5)	0.002
	>30	467 (49.7)	331 (53.3)	79 (47.6)	57 (37.5)	–
College or university	No	235 (25.0)	153 (24.6)	33 (19.9)	49 (32.2)	0.037
	Yes	704 (75.0)	468 (75.4)	133 (80.1)	103 (67.8)	–
Family monthly per capita income (Chinese yuan)	<5,000	424 (45.2)	277 (44.6)	73 (44.0)	74 (48.7)	0.680
	5,000–9,999	349 (37.2)	239 (38.5)	60 (36.1)	50 (32.9)	–
	≥10,000	166 (17.7)	105 (16.9)	33 (19.9)	28 (18.4)	–
Puerperium	Yes	202 (21.5)	143 (23.0)	33 (19.9)	26 (17.1)	0.240
	No	737 (78.5)	478 (77.0)	133 (80.1)	126 (82.9)	–
Primiparas	Yes	620 (66.0)	391 (63.0)	113 (68.1)	116 (76.3)	0.006
	No	319 (34.0)	230 (37.0)	53 (31.9)	36 (23.7)	–
BMI	Underweight or normal weight	593 (63.2)	390 (62.8)	108 (65.1)	95 (62.5)	0.946
	Overweight	264 (28.1)	174 (28.0)	46 (27.7)	44 (28.9)	–
	Obese	82 (8.7)	57 (9.2)	12 (7.2)	13 (8.6)	–
Physical activity	Low	343 (36.5)	226 (36.4)	60 (36.1)	57 (37.5)	0.978
	Medium	298 (31.7)	201 (32.4)	51 (30.7)	46 (30.3)	–
	High	298 (31.7)	194 (31.2)	55 (33.1)	49 (32.2)	–
Husband or partner support	Rarely	53 (5.6)	33 (5.3)	12 (7.2)	8 (5.3)	0.003
	Sometimes	211 (22.5)	119 (19.2)	54 (32.5)	38 (25.0)	–
	Always	675 (71.9)	469 (75.5)	100 (60.2)	106 (69.7)	–
Parent support	Rarely	61 (6.5)	35 (5.6)	16 (9.6)	10 (6.6)	0.066
	Sometimes	189 (20.1)	114 (18.4)	42 (25.3)	33 (21.7)	–
	Always	689 (73.4)	472 (76.0)	108 (65.1)	109 (71.7)	–
Cities	First-tier	187 (19.9)	122 (19.6)	36 (21.7)	29 (19.1)	0.697
	New first-tier	277 (29.5)	194 (31.2)	44 (26.5)	39 (25.7)	–
	Second-tier	283 (30.1)	184 (29.6)	48 (28.9)	51 (33.6)	–
	Third or fourth-tier	192 (20.4)	121 (19.5)	38 (22.9)	33 (21.7)	–
Regions	South	467 (49.7)	300 (48.3)	88 (53.0)	79 (52.0)	0.467
	North	472 (50.3)	321 (51.7)	78 (47.0)	73 (48.0)	–

a *Chi-square test*.

**Figure 1:**
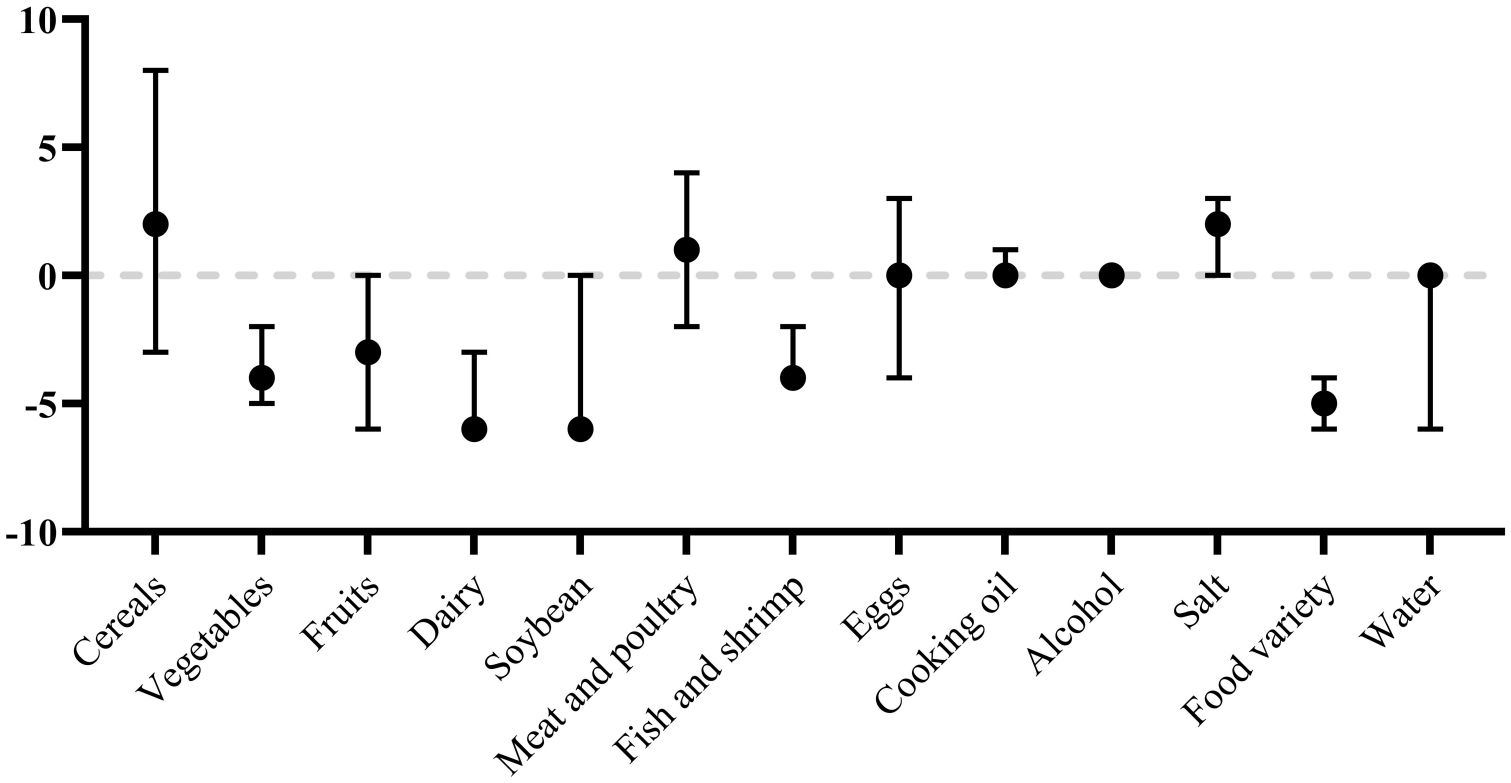
Scores [Median (25th, 75th)] for the DBI components.

Overall the proportion of participants meeting the recommended dietary intakes (score = 0) was ranged from 0.0 to 98.9%, with 0.0% for food variety, 7.8% for meat and poultry, 7.9% for dairy, 9.8% for eggs, 10.6% for vegetables, 11.0% for cereals, 18.6% for fish and shrimp, 26.0% for fruits, 28.4% for soybean, 31.1% for salt, 51.2% for water, 59.1% for cooking oil, and 98.9% for alcohol. As shown in Figure 1, the insufficient food intake of vegetables, fruits, dairy, soybean, and fish and shrimp were common. The median score of food variety was only −5.

**Figure 2:**
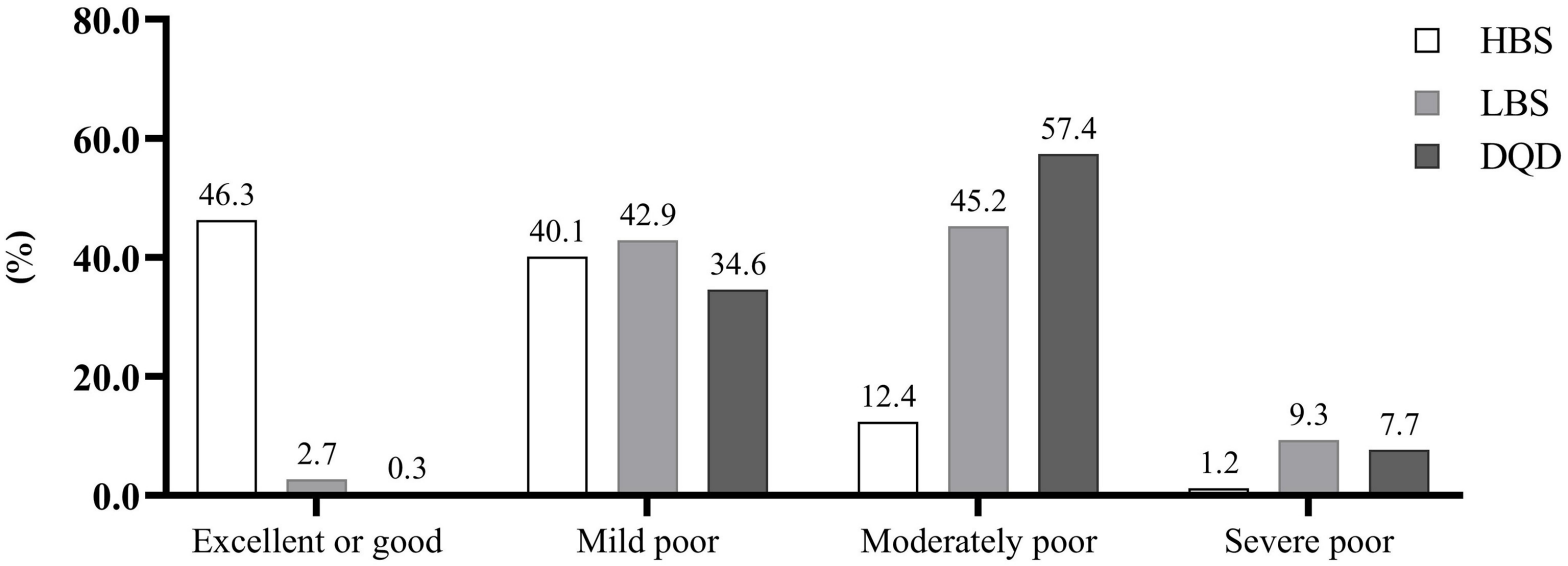
Distributions of HBS, LBS, and DQD among lactating women.

The median (25th, 75th) of overall HBS, LBS, and DQD was 9 (5, 14), 30 (25, 37), and 40 (34, 47), respectively. The sum of the proportions of HBS, LBS, and DQD in the poor level was as high as 53.7, 97.3, and 99.7%, respectively (Figure 2). As shown in Table 2, lactating women aged ≤ 30, with lower education level and family income tended to have higher LBS, and lactating women with lower education level and family income tended to have higher DQD.

**Table 2:** Comparison of HBS, LBS, and DQD [Median (25th, 75th)] by different characteristics of lactating women.

**Variables**		**HBS**	***P*^a^**	**LBS**	***P*^a^**	**DQD**	***P*^a^**
		**Median (25th, 75th)**		**Median (25th, 75th)**		**Median (25th, 75th)**	
Age (years)	≤ 30	9 (5, 14)	0.124	32 (25, 38)	0.023	41 (34, 48)	0.288
	>30	9 (6, 14)	–	30 (24, 35)	–	40 (34, 47)	–
College or university	No	9 (6, 14)	0.961	32 (27, 38)	0.001	43 (37, 49)	<0.001
	Yes	9 (5, 14)	–	30 (24, 36)	–	40 (34, 47)	–
Family monthly per capita income (Chinese yuan)	<5,000	9 (5, 14)	0.819	32 (25, 38)	0.007	42 (35, 49)	0.005
	5,000–9,999	9 (6, 14)	–	30 (24, 35)	–	40 (34, 46)	–
	≥10,000	9 (5, 15)	–	30 (23, 37)	–	40 (33, 46)	–
Puerperium	Yes	9 (5, 14)	0.456	31 (24, 36)	0.861	40 (34, 47)	0.713
	No	9 (5, 14)	–	30 (25, 37)	–	41 (34, 47)	–
Primiparas	Yes	9 (5, 14)	0.947	31 (24, 37)	0.349	41 (34, 48)	0.320
	No	9 (6, 14)	–	30 (25, 36)	–	40 (34, 46)	–
BMI	Underweight or normal weight	9 (6, 14)	0.612	30 (24, 37)	0.892	41 (34, 48)	0.986
	Overweight	9 (5, 13)	–	31 (25, 36)	–	40 (35, 47)	–
	Obese	9 (6, 13)	–	31 (23, 37)	–	40 (34, 47)	–
Physical activity	Low	9 (6, 15)	0.036	31 (25, 37)	0.813	42 (35, 48)	0.069
	Medium	9 (5, 12)	–	30 (24, 37)	–	40 (33, 46)	–
	High	9 (6, 14)	–	31 (24, 37)	–	40 (34, 48)	–
Husband or partner support	Rarely	9 (5, 14)	0.734	32 (26, 35)	0.299	41 (34, 47)	0.741
	Sometimes	9 (6, 14)	–	31 (26, 38)	–	41 (35, 47)	–
	Always	9 (5, 14)	–	30 (24, 37)	–	40 (34, 48)	–
Parent support	Rarely	9 (5, 14)	0.444	32 (26, 38)	0.353	41 (35, 48)	0.359
	Sometimes	9 (6, 14)	–	31 (25, 37)	–	42 (35, 48)	–
	Always	9 (5, 14)	–	30 (24, 36)	–	40 (34, 47)	–
Cities	First-tier	8 (5, 12)	<0.001	30 (24, 37)	0.065	39 (33, 46)	0.142
	New first-tier	9 (5, 14)	–	32 (25, 38)	–	42 (34, 48)	–
	Second-tier	10 (7, 15)	–	30 (24, 35)	–	40 (34, 47)	–
	Third or fourth-tier	9 (6, 13)	–	30 (24, 37)	–	40 (34, 46)	–
Regions	South	9 (5, 15)	0.089	30 (25, 36)	0.329	41 (34, 47)	0.730
	North	9 (5, 13)	–	31 (25, 37)	–	40 (34, 48)	–

a *Mann-Whitney U or Kruskal-Wallis H-test*.

**Figure 3:**
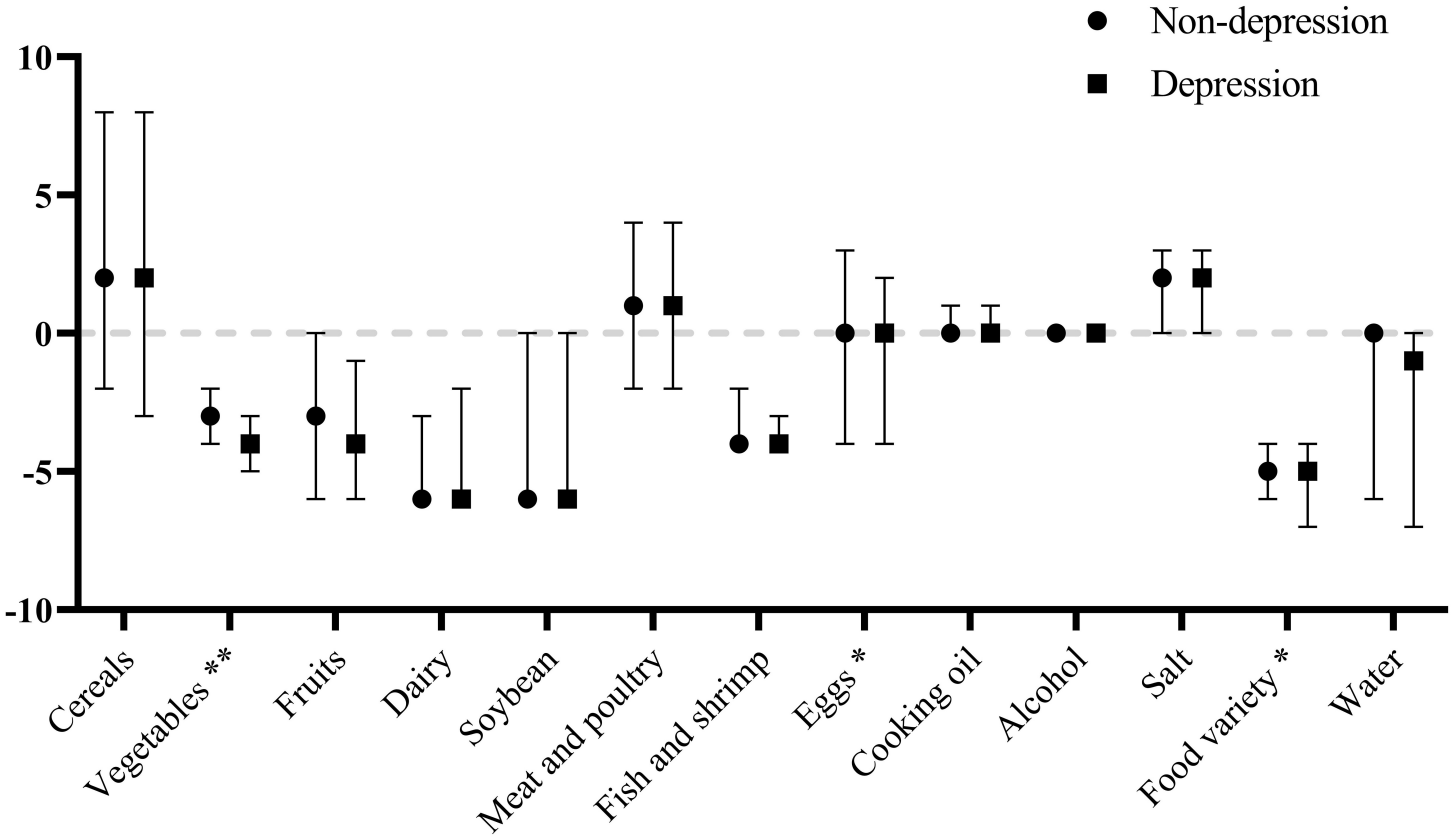
Comparison of scores [Median (25th, 75th)] for the DBI components by depression status. **P < 0.01; *P < 0.05.

As shown in Figure 3, depressed women tended to have a more inadequate intake of vegetables and have more insufficient food variety. However, depressed mothers tended to have a slightly more moderate intake of eggs. The correlations of total scores of EPDS with HBS, LBS, and DQD were −0.007 (*P* = 0.939), 0.064 (*P* = 0.048), and 0.065 (*P* = 0.047), respectively. As shown in Table 3, depressed women tended to have higher LBS and DQD.

**Table 3:** Comparison of HBS, LBS, and DQD [Median (25^th^, 75^th^)] by depression status.

	**Non-depression**	**Depression**	***P*^a^**
HBS	9 (5, 14)	9 (5, 14)	0.323
LBS	30 (24, 36)	31 (26, 39)	0.010
DQD	40 (34, 47)	42 (35, 49)	0.025

a *Mann-Whitney U-test*.

As shown in Table 4, a significant association was observed between LBS and postpartum depression; in addition, a significant association was observed between DQD and postpartum depression.

**Table 4:** Poisson regression analysis of associations of diet quality and postpartum depression.

	**PR (95%CI)^a^**	***P***	***P* for trend**
HBS
Q1	1.00 (Ref.)	–	0.666
Q2	0.99 (0.93, 1.05)	0.666	–
Q3	0.99 (0.93, 1.06)	0.824	–
Q4	0.98 (0.92, 1.05)	0.604	–
LBS
Q1	1.00 (Ref.)	–	0.043
Q2	1.05 (0.98, 1.12)	0.154	–
Q3	1.02 (0.96, 1.08)	0.631	–
Q4	1.08 (1.01, 1.15)	0.016	–
DQD
Q1	1.00 (Ref.)	–	0.036
Q2	0.99 (0.93, 1.06)	0.805	–
Q3	1.00 (0.94, 1.07)	0.956	–
Q4	1.07 (1.00, 1.14)	0.040	–

a *Adjusted for maternal age, education, primiparas, and husband or partner support*.

## Discussion

To our knowledge, it is the first study to assess the association of dietary quality and postpartum depression of Chinese lactating women in the first year postpartum based on the DBI. We found that nearly one-third of Chinese lactating women in the first year postpartum suffered from depressive symptoms. Those with younger age, lower education, being primiparous, and with less support from husband or partner were more vulnerable. The dietary quality of lactating women was poor, the intake of vegetables, fruits, dairy, soybean, and fish and shrimp was insufficient, and the food variety was very limited. Depressed women had less vegetable intake and had more insufficient food variety. An imbalanced diet was associated with an increased risk of postpartum depression.

Postpartum depression is a mood disorder associated with childbirth, usually develops between a week and a month after delivery. Puerperium is considered as a period of the high prevalence of mental disorder illnesses in women (30), but in this study, no significant difference was observed between women in puerperium and after puerperium. Postpartum depression can be spontaneously alleviated within 3–6 months, but some depressed mothers still have symptoms at 1 year, with increased negative effects on both maternal and children's health and well-being (31). Some researchers suggest the assessment period of postpartum depression should be extended to 12 months postpartum (32). Further, although the National Health Commission (NHC) of China has planned that introducing screening for depression during pregnancy and childbirth to routine antenatal care and postpartum visits, one study has found that the majority of symptom-positive women during the perinatal period do not accept a subsequent referral to mental health services (33). Depressed lactating women who are recommended to take an antidepressant may concern that the drugs have a possible effect on their infants via breast milk (34). Therefore, understanding the associations between dietary quality and postpartum depression may have some certain meanings.

We found younger mothers tended to have a higher risk of postpartum depression, which was in line with previous researches (35, 36). One possible reason was that younger mothers might not be fully prepared for the new maternal role. However, the associations of maternal age and risk of depression were still inconclusive. A study has reported that women of advanced maternal age have significantly higher rates of depression than younger women (37). Another study has reported that women's risk for depression increases with age, and age 30 is a critical point (38). Considering the inconsistencies in the existing studies, there is a need for further longitudinal and qualitative studies to understand the associations between maternal age and postpartum depression. We also found less educated women had a higher proportion of postpartum depression, consistent with many studies (39, 40). Further, primiparas had a higher proportion of postpartum depression. Being a first-time mother was a huge challenge, and it is difficult for primiparas to adapt to their new role (41, 42). Lack of husband or partner support was a strong risk factor on postpartum depression (43, 44), and we also observed similar results.

We did not observe the associations between family monthly per capita income and postpartum depression, inconsistent with previous studies (45, 46). One of the possible explanations is that the data were based on an investigation in urban areas, and the surveyed women have a relatively higher economic level. Future studies could include women from different socioeconomic backgrounds to better understand the associations between family income and postpartum depression among Chinese lactating women. We also did not observe the associations between BMI and postpartum depression. Several biological mechanisms have been hypothesized underlying the association between weight and mental health, such as inflammation, the dysregulation of the hypothalamus-pituitary-adrenal axis, and metabolic changes (47), but the association between BMI and postpartum depression are mixed in previous studies (48, 49). Since the lactation period is one of substantial weight change and retention for women, the weight information provided by single-point measurement would be limited to some extent, especially for women who had just given birth. Future studies could include information about dynamic changes in body weight during postpartum to understand the associations between BMI and postpartum depression. Although we did not observe the associations between physical activity and postpartum depression, the benefits of physical activity during the postpartum period to postpartum depression have been proved in many types of research, consistent with those for depression among the general population (50, 51). Mental and physical health are interdependent, and activities which are started after prior consultation with a specialist and proper instruction are recommended (51).

We found depressed women had less vegetable consumption. Higher vegetable consumption, particularly yellow/orange/red and leafy green vegetables, may help to lower depressive symptoms (52, 53), and the possible explanation is certain nutrients found in vegetables, such as vitamins, phytochemicals, minerals, fiber, and amino acids (52). We also found depressed women had more insufficient food variety. Based on our data, a decreased food variety was associated with lower consumption of vegetables, fruit, dairy, and fish and shrimp. All these foods provide our bodies with essential nutrients, such as vitamins, phytochemicals, polyunsaturated fatty acids, and proteins. One study has reported that with an increase in food diversity, the dietary pattern of the individuals could become more similar to the Mediterranean dietary pattern (54), which is inversely associated with many mental health problems, including depression, anxiety, and psychological distress (55). Further, higher LBS and DQD were associated with an increased risk of postpartum depression. Based on DBI calculating method, a score of 0 is in full compliance with the dietary guidelines, and the farther the score is from 0, the lower the quality of the diet. Higher LBS and DQD indicated more insufficient intake and a more imbalanced diet. The negative association between adherence to dietary guidelines and mental disorders has been reported among Western countries (56, 57). This study added related evidence in China, where lifestyle components of lactating women are significantly different from those in Western countries. This favorable association of poor diet quality with postpartum depression may have been attributed to some components of DBI, including vegetables, fruit, dairy, and fish and shrimp, and the cumulatively diverse neuro-psychological effects of all diets. One study has reported the inverse association of Alternative Healthy Eating Index (AHEI) with depression and suggested this inverse association has been generated by the cumulative effects of all components of AHEI rather than an individual nutrient or food group (58). The anti-depression effect of nutrients is cumulative, and a balanced diet includes multiple components and complex interactions between dietary components and nutrients and their synergistic effects (56, 59). Our results highlighted the importance of maintaining the proper dietary balance on the decreased risk of postpartum depression.

Although the current study improves a broader understanding of dietary quality and postpartum depression, several limitations should be considered. First, because the analyses were carried out using cross-sectional data, no causal interpretations can be made. Notably, there may be a bidirectional association in that depressive symptoms and diet quality could be dependent upon one another. More prospective and longitudinal studies are needed. Second, a note of caution was due regarding the extrapolation of our results, in that it was a convenience sample in urban areas of China. Future studies with more representative samples are needed to confirm our results. Third, the DBI score was calculated using a single 24-h recall, which may not reflect usual dietary intake (60). In addition, recall bias cannot be excluded. In our fieldwork, we used a simple semi-quantitative food frequency questionnaire (SFFQ) to obtain participants' average daily food consumption in the past month at the same time. We calculated Spearman's correlation coefficient between 24HDR-derived and SFFQ-derived food groups, and all *P*-values were < 0.01. The correlation coefficient between 24HDR-derived and SFFQ-derived data may help demonstrate the interrelations between various food groups assessed by different dietary assessment tools. Future studies should further explore and optimize the dietary assessments to obtain more adequate habitual food consumption data (61, 62). Fourth, information on some variables, such as marital satisfaction, domestic violence, depression history, and other mental disorders, were not available for our study. Although we have adjusted for a series of potentially confounding covariates, we cannot rule out the possibility of residual confounding.

## Conclusion

In our studied population, nearly one-third of lactating women in the first year postpartum suffered from depressive symptoms. Depressed women had less vegetable intake and had more insufficient food variety. An imbalanced diet was associated with an increased risk of postpartum depression.

## Data Availability Statement

The raw data supporting the conclusions of this article will be made available by the authors, without undue reservation.

## Ethics Statement

The studies involving human participants were reviewed and approved by the Medical Ethics Research Board of Peking University (NO. IRB00001052-19045). The patients/participants provided their written informed consent to participate in this study.

## Author Contributions

AZ, IS, PW, and YZ: conceptualization. CY, ZR, and JZ: formal analysis. CY, AZ, HL, ZR, and JZ: investigation and methodology. CY: writing—original draft. AZ, HL, IS, PW, and YZ: writing—review and editing. All authors contributed to the article and approved the submitted version.

## Conflict of Interest

IS and HL are employed by Inner Mongolia Yili Industrial Group Co. Ltd. This study received funding from Inner Mongolia Yili Industrial Group Co. Ltd. The funder had the following involvement with the study: conceptualization, investigation, methodology, and writing—review and editing.

The remaining authors declare that the research was conducted in the absence of any commercial or financial relationships that could be construed as a potential conflict of interest.

## Publisher's Note

All claims expressed in this article are solely those of the authors and do not necessarily represent those of their affiliated organizations, or those of the publisher, the editors and the reviewers. Any product that may be evaluated in this article, or claim that may be made by its manufacturer, is not guaranteed or endorsed by the publisher.

## Supplementary Material

Supplementary Table 1 The DBI calculating methods in this study.

## References

[1] Herba CM Glover V Ramchandani PG Rondon MB. Maternal depression and mental health in early childhood: an examination of underlying mechanisms in low-income and middle-income countries. Lancet Psychiatry. (2016) 3:983–92. 10.1016/S2215-0366(16)30148-1 27650772

[2] Gelaye B Rondon MB Araya R Williams MA. Epidemiology of maternal depression, risk factors, and child outcomes in low-income and middle-income countries. Lancet Psychiatry. (2016) 3:973–82. 10.1016/S2215-0366(16)30284-X 27650773 PMC5155709

[3] Li Y Zhao Q Cross WM Chen J Qin C Sun M. Assessing the quality of mobile applications targeting postpartum depression in China. Int J Mental Health Nurs. (2020) 29:772–85. 10.1111/inm.12713 32223070

[4] Nisar A Yin J Waqas A Bai X Wang D Rahman A. Prevalence of perinatal depression and its determinants in Mainland China: a systematic review and meta-analysis. J Affect Disord. (2020) 277:1022–37. 10.1016/j.jad.2020.07.046 33065811

[5] Payne JL Maguire J. Pathophysiological mechanisms implicated in postpartum depression. Front Neuroendocrinol. (2019) 52:165–80. 10.1016/j.yfrne.2018.12.001 30552910 PMC6370514

[6] O'Hara MW McCabe JE. Postpartum depression: current status and future directions. Annu Rev Clin Psychol. (2013) 9:379–407. 10.1146/annurev-clinpsy-050212-185612 23394227

[7] Marx W Moseley G Berk M Jacka F. Nutritional psychiatry: the present state of the evidence. Proc Nutr Soc. (2017) 76:427–36. 10.1017/S0029665117002026 28942748

[8] Opie RS Uldrich AC Ball K. Maternal postpartum diet and postpartum depression: a systematic review. Matern Child Health J. (2020) 24:966–78. 10.1007/s10995-020-02949-9 32367245

[9] Jacka FN Pasco JA Mykletun A Williams LJ Hodge AM O'Reilly SL. Association of Western and traditional diets with depression and anxiety in women. Am J Psychiatry. (2010) 167:305–11. 10.1176/appi.ajp.2009.09060881 20048020

[10] Nathanson R Hill B Skouteris H Bailey C. Antenatal diet and postpartum depressive symptoms: a prospective study. Midwifery. (2018) 62:69–76. 10.1016/j.midw.2018.03.015 29655007

[11] Pollak MJ Milte CM van der Pligt P Teychenne M. Total physical activity but not diet quality associated with postnatal depressive symptoms amongst women living in socioeconomically disadvantaged neighborhoods. Nutr Res. (2019) 68:54–61. 10.1016/j.nutres.2019.05.009 31421393

[12] Baskin R Hill B Jacka FN O'Neil A Skouteris H. The association between diet quality and mental health during the perinatal period. A systematic review. Appetite. (2015) 91:41–7. 10.1016/j.appet.2015.03.017 25814192

[13] Zheng L Sun J Yu X Zhang D. Ultra-processed food is positively associated with depressive symptoms among United States adults. Front Nutr. (2020) 7:600449. 10.3389/fnut.2020.600449 33385006 PMC7770142

[14] Zheng X Chen J Xie T Xia Z Loo WTY Lao L. Relationship between Chinese medicine dietary patterns and the incidence of breast cancer in Chinese women in Hong Kong: a retrospective cross-sectional survey. Chin Med. (2017) 12:17. 10.1186/s13020-017-0138-9 28670332 PMC5492296

[15] Liu Y Hu J Chen X Yu Y Bai J. Effects of a health education program targeted to Chinese women adhering to their cultural practice of doing the month: a randomized controlled trial. Midwifery. (2020) 90:102796. 10.1016/j.midw.2020.102796 32726727

[16] Ding G Niu L Vinturache A Zhang J Lu M Gao Y. “Doing the month” and postpartum depression among Chinese women: a Shanghai prospective cohort study. Women Birth. (2020) 33:e151–8. 10.1016/j.wombi.2019.04.004 31060983

[17] Liu N Mao L Sun X Liu L Yao P Chen B. The effect of health and nutrition education intervention on women's postpartum beliefs and practices: a randomized controlled trial. BMC Public Health. (2009) 9:45. 10.1186/1471-2458-9-45 19183504 PMC2640472

[18] Huang Y Wang X Yang Y Qu X Wang A Huang X. The role of education in maternal depressive symptoms among different ethnic groups: a cross-sectional study in rural western China. J Affect Disord. (2020) 262:359–65. 10.1016/j.jad.2019.11.022 31735406

[19] Lau Y Wang Y Yin L Chan KS Guo X. Validation of the Mainland Chinese version of the Edinburgh Postnatal Depression Scale in Chengdu mothers. Int J Nurs Stud. (2010) 47:1139–51. 10.1016/j.ijnurstu.2010.02.005 20219196

[20] Ding Y Indayati W Basnet TB Li F Luo H Pan H. Dietary intake in lactating mothers in China 2018: report of a survey. Nutr J. (2020) 19:72. 10.1186/s12937-020-00589-x 32664937 PMC7362564

[21] Yang YY. China Food Composition. 2nd ed. Beijing: Beijing Medical University Press (2009).

[22] He D Qiao Y Xiong S Liu S Ke C Shen Y. Association between dietary quality and prediabetes based on the diet balance index. Sci Rep. (2020) 10:3190. 10.1038/s41598-020-60153-9 32081975 PMC7035297

[23] Han A Sun T Ming J Chai L Liao X. Are the Chinese moving toward a healthy diet? Evidence from Macro data from 1961 to 2017. Int J Environ Res Public Health. (2020) 17:5294. 10.3390/ijerph17155294 32717812 PMC7432933

[24] Yuna H Yuehui F Juan X. [Update of the Chinese diet balance index: DBI_16]. Acta Nutrimenta Sinica. (2018) 40:526–30. 10.3969/j.issn.0512-7955.2018.06.002

[25] Yuna H Fengying Z Xiaoguang Y Keyou G. [The Chinese diet balance index revised]. Acta Nutrimenta Sinica. (2009) 31:532–6. 10.3321/j.issn:0512-7955.2009.06.00325567009

[26] Yuna H Fengying Z Keyou G. [Approaching Chinese diet balance index]. J Hyg Res. 2005:208–11. 10.3969/j.issn.1000-8020.2005.02.025 15952666

[27] Su X Zhu W Li N Sun J Zhu Y Liu T. Adjusting DBI-2016 to dietary balance index for Chinese maternal women and assessing the association between maternal dietary quality and postpartum weight retention: a longitudinal study. PLoS ONE. (2020) 15:e0237225. 10.1371/journal.pone.0237225 32817619 PMC7444517

[28] Chen C Lu FC. The guidelines for prevention and control of overweight and obesity in Chinese adults. Biomed Environ Sci. (2004) 17(Suppl.):1–36. 10.1111/j.1365-2028.2008.00991.x 15807475

[29] Fan M Lyu J He P. [Chinese guidelines for data processing and analysis concerning the International Physical Activity Questionnaire]. Zhonghua Liu Xing Bing Xue Za Zhi. (2014) 35:961–4. 10.3760/cma.j.issn.0254-6450.2014.08.019 25376692

[30] Silverman ME Reichenberg A Savitz DA Cnattingius S Lichtenstein P Hultman CM. The risk factors for postpartum depression: a population-based study. Depress Anxiety. (2017) 34:178–87. 10.1002/da.22597 28098957 PMC5462547

[31] Craig M Howard L. Postnatal depression. BMJ Clin Evid. (2009) 2009:1407. 10.1016/0140-6736(93)92263-S PMC2907780 19445768

[32] Gaynes BN Gavin N Meltzer-Brody S Lohr KN Swinson T Gartlehner G. Perinatal depression: prevalence, screening accuracy, and screening outcomes. Evid Report Technol Assess. 2005:1–8. 10.1037/e439372005-001 15760246 PMC4780910

[33] Gong W Jin X Cheng KK Caine ED Lehman R Xu DR. Chinese women's acceptance and uptake of referral after screening for perinatal depression. Int J Environ Res Public Health. (2020) 17:8686. 10.3390/ijerph17228686 33238480 PMC7700456

[34] Rampono J Kristensen JH Hackett LP Paech M Kohan R Ilett KF. Citalopram and demethylcitalopram in human milk; distribution, excretion and effects in breast fed infants. Br J Clin Pharmacol. (2000) 50:263–8. 10.1046/j.1365-2125.2000.00253.x 10971311 PMC2014979

[35] Liu S Yan Y Gao X Xiang S Sha T Zeng G. Risk factors for postpartum depression among Chinese women: path model analysis. BMC Pregnancy Childbirth. (2017) 17:133. 10.1186/s12884-017-1320-x 28464884 PMC5414210

[36] Khalifa DS Glavin K Bjertness E Lien L. Determinants of postnatal depression in Sudanese women at 3 months postpartum: a cross-sectional study. BMJ Open. (2016) 6:e009443. 10.1136/bmjopen-2015-009443 26966055 PMC4800153

[37] Muraca GM Joseph KS. The association between maternal age and depression. J Obstetr Gynaecol Can. (2014) 36:803–10. 10.1016/S1701-2163(15)30482-5 25222359

[38] Luke S Salihu HM Alio AP Mbah AK Jeffers D Berry EL. Risk factors for major antenatal depression among low-income African American women. J Womens Health (2002). (2009) 18:1841–6. 10.1089/jwh.2008.1261 19951220

[39] Matsumura K Hamazaki K Tsuchida A Kasamatsu H Inadera H. Education level and risk of postpartum depression: results from the Japan Environment and Children's Study (JECS). BMC Psychiatry. (2019) 19:419. 10.1186/s12888-019-2401-3 31882000 PMC6935197

[40] Do TKL Nguyen TTH Pham TTH. Postpartum depression and risk factors among vietnamese women. BioMed Res Int. (2018) 2018:4028913. 10.1155/2018/4028913 30320133 PMC6167583

[41] Zhou C Zheng W Yuan Q Zhang B Chen H Wang W. Associations between social capital and maternal depression: results from a follow-up study in China. BMC Pregnancy Childbirth. (2018) 18:45. 10.1186/s12884-018-1673-9 29394914 PMC5797398

[42] Wang Q Zhang Y Li X Ye Z Huang L Zhang Y. Exploring maternal self-efficacy of first-time mothers among rural-to-urban floating women: a quantitative longitudinal study in China. Int J Environ Res Public Health. (2021) 18:2793. 10.3390/ijerph18062793 33801851 PMC8001710

[43] Li Q Yang S Xie M Wu X Huang L Ruan W. Impact of some social and clinical factors on the development of postpartum depression in Chinese women. BMC Pregnancy Childbirth. (2020) 20:226. 10.1186/s12884-020-02906-y 32299376 PMC7164157

[44] Upadhyay RP Chowdhury R Aslyeh S Sarkar K Singh SK Sinha B. Postpartum depression in India: a systematic review and meta-analysis. Bull World Health Organization. (2017) 95:706–17c. 10.2471/BLT.17.192237 29147043 PMC5689195

[45] Leung BM Letourneau NL Giesbrecht GF Ntanda H Hart M. Predictors of postpartum depression in partnered mothers and fathers from a longitudinal cohort. Community Mental Health J. (2017) 53:420–31. 10.1007/s10597-016-0060-0 27826783

[46] Gebregziabher NK Netsereab TB Fessaha YG Alaza FA Ghebrehiwet NK Sium AH. Prevalence and associated factors of postpartum depression among postpartum mothers in central region, Eritrea: a health facility based survey. BMC Public Health. (2020) 20:1614. 10.1186/s12889-020-09676-4 33109137 PMC7590801

[47] Silverman ME Smith L Lichtenstein P Reichenberg A Sandin S. The association between body mass index and postpartum depression: a population-based study. J Affect Disord. (2018) 240:193–8. 10.1016/j.jad.2018.07.063 30077160

[48] Sparling TM Waid JL Wendt AS Gabrysch S. Depression among women of reproductive age in rural Bangladesh is linked to food security, diets and nutrition. Public Health Nutr. (2020) 23:660–73. 10.1017/S1368980019003495 31915095 PMC7058425

[49] Carter AS Baker CW Brownell KD. Body mass index, eating attitudes, and symptoms of depression and anxiety in pregnancy and the postpartum period. Psychosom Med. (2000) 62:264–70. 10.1097/00006842-200003000-00019 10772407

[50] Dipietro L Evenson KR Bloodgood B Sprow K Troiano RP Piercy KL. Benefits of physical activity during pregnancy and postpartum: an umbrella review. Med Sci Sports Exerc. (2019) 51:1292–302. 10.1249/MSS.0000000000001941 31095086 PMC6527310

[51] Kołomańska-Bogucka D Mazur-Bialy AI. Physical activity and the occurrence of postnatal depression-a systematic review. Medicina. (2019) 55:560. 10.3390/medicina55090560 31480778 PMC6780177

[52] Radavelli-Bagatini S Anokye R Bondonno NP Sim M Bondonno CP Stanley MJ. Association of habitual intake of fruits and vegetables with depressive symptoms: the AusDiab study. Eur J Nutr. (2021). 10.1007/s00394-021-02532-0. [Epub ahead of print]. 33778912

[53] Saghafian F Malmir H Saneei P Milajerdi A Larijani B Esmaillzadeh A. Fruit and vegetable consumption and risk of depression: accumulative evidence from an updated systematic review and meta-analysis of epidemiological studies. Br J Nutr. (2018) 119:1087–101. 10.1017/S0007114518000697 29759102

[54] Poorrezaeian M Siassi F Milajerdi A Qorbani M Karimi J Sohrabi-Kabi R. Depression is related to dietary diversity score in women: a cross-sectional study from a developing country. Ann Gen Psychiatry. (2017) 16:39. 10.1186/s12991-017-0162-2 29176995 PMC5689184

[55] Sadeghi O Keshteli AH Afshar H Esmaillzadeh A Adibi P. Adherence to Mediterranean dietary pattern is inversely associated with depression, anxiety and psychological distress. Nutr Neurosci. (2021) 24:248–59. 10.1080/1028415X.2019.1620425 31185883

[56] Wu PY Lin MY Tsai PS. Alternate healthy eating index and risk of depression: a meta-analysis and systemematic review. Nutr Neurosci. (2020) 23:101–9. 10.1080/1028415X.2018.1477424 29804517

[57] Deierlein AL Ghassabian A Kahn LG Afanasyeva Y Mehta-Lee SS Brubaker SG. Dietary quality and sociodemographic and health behavior characteristics among pregnant women participating in the New York University children's health and environment study. Front Nutr. (2021) 8:639425. 10.3389/fnut.2021.639425 33898496 PMC8062781

[58] Saneei P Hajishafiee M Keshteli AH Afshar H Esmaillzadeh A Adibi P. Adherence to Alternative Healthy Eating Index in relation to depression and anxiety in Iranian adults. Br J Nutr. (2016) 116:335–42. 10.1017/S0007114516001926 27188471

[59] Gianfredi V Koster A Odone A Amerio A Signorelli C Schaper NC. Associations of dietary patterns with incident depression: the maastricht study. Nutrients. (2021) 131034. 10.3390/nu13031034 33806882 PMC8004955

[60] Verly E Jr Oliveira DC Fisberg RM Marchioni DM. Performance of statistical methods to correct food intake distribution: comparison between observed and estimated usual intake. Br J Nutr. (2016) 116:897–903. 10.1017/S0007114516002725 27523187

[61] Verly E Jr Castro MA Fisberg RM Marchioni DM. Precision of usual food intake estimates according to the percentage of individuals with a second dietary measurement. J Acad Nutr Dietetics. (2012) 112:1015–20. 10.1016/j.jand.2012.03.028 22889632

[62] Verly Junior E Cesar CL Fisberg RM Marchioni DM. [Within-person variance of the energy and nutrient intake in adolescents: data adjustment in epidemiological studies]. Revista brasileira de epidemiologia. (2013) 16:170–7. 10.1590/S1415-790X2013000100016 23681333

