# Supplementary material for: Association Between Dietary Quality and Postpartum Depression in Lactating Women: A Cross-Sectional Survey in Urban China
Source: Front Nutr. 2021 Aug 26;8:705353. doi: 10.3389/fnut.2021.705353 (PMC8427431; doi:10.3389/fnut.2021.705353)
Supplement: Supplementary Table 1 — The DBI calculating methods in this study. [file Table_1.DOCX]

Supplementary Material

**Table S1.** The DBI calculating methods in this study.

| **Components** | **Score range** | **Energy (kcal/day)** | | | | | | |
| --- | --- | --- | --- | --- | --- | --- | --- | --- |
|  |  | 1800 | 2000 | 2200 | 2400 | 2600 | 2800 | 3000 |
| cereals | -12~12 | <35g = -12 | <5g = -12 | <30g = -12 | 0g = -12 | <50g = -12 | <75g = -12 | <100g = -12 |
|  |  | 200~250g = 0 | 225~275g = 0 | 250~300g=0 | 275~325g = 0 | 325~375g = 0 | 350~400g = 0 | 375~425g = 0 |
|  |  | >415g = 12 | >495g = 12 | >520g = 12 | >600g = 12 | >650g = 12 | >675g = 12 | >700g = 12 |
|  |  | Score increased by 1 with intake amount increased by 15 g | Score increased by 1 with intake amount increased by 20 g | Score increased by 1 with intake amount increased by 20 g | Score increased by 1 with intake amount increased by 25 g | Score increased by 1 with intake amount increased by 25 g | Score increased by 1 with intake amount increased by 25 g | Score increased by 1 with intake amount increased by 25 g |
| vegetables | -6~0 | ≥400g = 0 | ≥450g = 0 |  | ≥500g = 0 |  |  | ≥600g = 0 |
|  |  | 320~399g = -1 | 360~449g = -1 |  | 400~499g = -1 |  |  | 480~599g =-1 |
|  |  | 0g = -6 | 0g = -6 |  | 0g = -6 |  |  | 0g = -6 |
|  |  | Score decreased by 1 with intake amount decreased by 80 g | Score decreased by 1 with intake amount decreased by 90 g |  | Score decreased by 1 with intake amount decreased by 100 g |  |  | Score decreased by 1 with intake amount decreased by 120 g |
| fruits | -6~0 | ≥200g = 0 | ≥300g = 0 |  | ≥350g = 0 |  | ≥400g = 0 |  |
|  |  | 160~199g = -1 | 240~299g = -1 |  | 280~349g = -1 |  | 320~399g = -1 |  |
|  |  | 0g = -6 | 0g = -6 |  | 0g = -6 |  | 0g = -6 |  |
|  |  | Score decreased by 1 with intake amount decreased by 40 g | Score decreased by 1 with intake amount decreased by 60 g |  | Score decreased by 1 with intake amount decreased by 70 g |  | Score decreased by 1 with intake amount decreased by 80 g |  |
| dairy | -6~0 | ≥400g = 0 |  |  |  |  |  |  |
|  |  | 0g = -6 |  |  |  |  |  |  |
|  |  | Score decreased by 1 with intake amount decreased by 80 g |  |  |  |  |  |  |
| soybean | -6~0 | ≥15g = 0 |  | ≥25g = 0 |  |  |  |  |
|  |  | 0g = -6 |  | 0g = -6 |  |  |  |  |
|  |  | Score decreased by 1 with intake amount decreased by 3 g |  | Score decreased by 1 with intake amount decreased by 5 g |  |  |  |  |
| meat and poultry | -4~4 | 0g = -4 |  | 0g = -4 |  |  | 0g = -4 |  |
|  |  | 1~20g = -3 |  | 1~25g = -3 |  |  | 1~35g = -3 |  |
|  |  | 21~40g = -2 |  | 26~50g = -2 |  |  | 36~70g = -2 |  |
|  |  | 41~50g = -1 |  | 51~75g = -1 |  |  | 71~100g = -1 |  |
|  |  | 51~70g = 0 |  | 76~95g = 0 |  |  | 101~120g = 0 |  |
|  |  | 71~90g = 1 |  | 96~120g = 1 |  |  | 121~155g = 1 |  |
|  |  | 91~110g=2 |  | 121~145g=2 |  |  | 156~190g=2 |  |
|  |  | 111~130g = 3 |  | 146~170g = 3 |  |  | 191~225g = 3 |  |
|  |  | >130g = 4 |  | >170g = 4 |  |  | >225g = 4 |  |
| fish and shrimp | -4~0 | 0g = -4 |  | 0g = -4 |  |  | 0g = -4 | 0g = -4 |
|  |  | 1~20g = -3 |  | 1~30g = -3 |  |  | 1~35g = -3 | 1~45g = -3 |
|  |  | 21~40g = -2 |  | 31~60g = -2 |  |  | 36~70g = -2 | 46~90g = -2 |
|  |  | 41~60g = -1 |  | 61~85g = -1 |  |  | 71~110g = -1 | 91~135g = -1 |
|  |  | >60g = 0 |  | >85g = 0 |  |  | >110g = 0 | >135g = 0 |
| eggs | -4~4 | 0g = -4 |  | 0g = -4 |  |  |  |  |
|  |  | 1~10g = -3 |  | 1~15g = -3 |  |  |  |  |
|  |  | 11~20g = -2 |  | 16~30g = -2 |  |  |  |  |
|  |  | 21~30g = -1 |  | 31~45g = -1 |  |  |  |  |
|  |  | 31~50g = 0 |  | 46~55g = 0 |  |  |  |  |
|  |  | 51~60g = 1 |  | 56~70g = 1 |  |  |  |  |
|  |  | 61~70g=2 |  | 71~85g=2 |  |  |  |  |
|  |  | 71~80g = 3 |  | 86~100g = 3 |  |  |  |  |
|  |  | >80g = 4 |  | >100g = 4 |  |  |  |  |
| cooking oil | 0~6 | ≤25g = 0 |  |  | ≤30g = 0 |  |  | ≤35g = 0 |
|  |  | 26~30g = 1 |  |  | 31~35g = 1 |  |  | 36~40g = 1 |
|  |  | >50g = 6 |  |  | >55g = 6 |  |  | >60g = 6 |
|  |  | Score increased by 1 with intake amount increased by 5 g |  |  | Score increased by 1 with intake amount increased by 5 g |  |  | Score increased by 1 with intake amount increased by 5 g |
| alcohol | 0~6 | 0g = 0 |  |  |  |  |  |  |
|  |  | >50g = 6 |  |  |  |  |  |  |
|  |  | Score increased by 1 with intake amount increased by 10 g |  |  |  |  |  |  |
| salt | 0~6 | ≤6g = 0 |  |  |  |  |  |  |
|  |  | 6~7g=1 |  |  |  |  |  |  |
|  |  | >16g = 6 |  |  |  |  |  |  |
|  |  | Score increased by 1 with intake amount increased by 2 g |  |  |  |  |  |  |
| food variety ^a^ | -12~0 | ≥ 12 kinds of food = 0 |  |  |  |  |  |  |
|  |  | Score decreased by 1 with food variety decreased by 1 |  |  |  |  |  |  |
| water | -12~0 | ≥1400ml=0 |  |  |  |  |  |  |
|  |  | Score decreased by 1 with water intake decreased by 100 ml |  |  |  |  |  |  |

^a^ Food variety: 1) Rice and products; 2) Wheat and products; 3) Corn, coarse grains and products, Starchy roots and products; 4) Dark-colored vegetables; 5) Light-colored vegetables; 6) Fruits; 7) Soybean and products; 8) Dairy products; 9) Meat; 10) Poultry; 11) Eggs; 12) Fish and shrimp.
